# Multitarget high-definition transcranial direct current stimulation improves response inhibition more than single-target high-definition transcranial direct current stimulation in healthy participants

**DOI:** 10.3389/fnins.2022.905247

**Published:** 2022-07-29

**Authors:** Zhihua Guo, Yue Gong, Hongliang Lu, Rui Qiu, Xinlu Wang, Xia Zhu, Xuqun You

**Affiliations:** ^1^Department of Military Medical Psychology, Air Force Medical University, Xi’an, China; ^2^School of Psychology, Shaanxi Normal University, Xi’an, China

**Keywords:** high-definition transcranial direct current stimulation (HD-tDCS), response inhibition, right inferior frontal gyrus (rIFG), pre-supplementary motor area (pre-SMA), fNIRS

## Abstract

Prior studies have focused on single-target anodal transcranial direct current stimulation (tDCS) over the right inferior frontal gyrus (rIFG) or pre-supplementary motor area (pre-SMA) to improve response inhibition in healthy individuals. However, the results are contradictory and the effect of multitarget anodal stimulation over both brain regions has never been investigated. The present study aimed to investigate the behavioral and neurophysiological effects of different forms of anodal high-definition tDCS (HD-tDCS) on improving response inhibition, including HD-tDCS over the rIFG or pre-SMA and multitarget HD-tDCS over both areas. Ninety-two healthy participants were randomly assigned to receive single-session (20 min) anodal HD-tDCS over rIFG + pre-SMA, rIFG, pre-SMA, or sham stimulation. Before and immediately after tDCS intervention, participants completed a stop-signal task (SST) and a go/nogo task (GNG). Their cortical activity was recorded using functional near-infrared spectroscopy (fNIRS) during the go/nogo task. The results showed multitarget stimulation produced a significant reduction in stop-signal reaction time (SSRT) relative to baseline. The pre-to-post SSRT change was not significant for rIFG, pre-SMA, or sham stimulation. Further analyses revealed multitarget HD-tDCS significantly decreased SSRT in both the high-performance and low-performance subgroups compared with the rIFG condition which decreased SSRT only in the low-performance subgroup. Only the multitarget condition significantly improved neural efficiency as indexed by lower △oxy-Hb after stimulation. In conclusion, the present study provides important preliminary evidence that multitarget HD-tDCS is a promising avenue to improve stimulation efficacy, establishing a more effective montage to enhance response inhibition relative to the commonly used single-target stimulation.

## Introduction

Response inhibition refers to the ability to inhibit inappropriate or irrelevant responses so that one can make flexible and goal-directed behavioral responses to changes in the environment, which is an important part of executive function (Verbruggen and Logan, 2008, 2009; Diamond, 2013). Response inhibition is involved in many everyday activities, such as a driver stopping from pressing the accelerator in order to not hit a pedestrian. Prior studies have shown that response inhibition is related to decision-making (Xu et al., 2020), working memory (Alderson et al., 2017), impulse control (Mayer et al., 2020), etc. Additionally, many psychiatric disorders are associated with deficits in response inhibition (Hughes et al., 2012; Steele et al., 2014; van Rooij et al., 2015; Gowda et al., 2019; Alizadehgoradel et al., 2020; Sun et al., 2021). In recent years, studies have increasingly focused on the neural substrates of response inhibition and have demonstrated that it is based on the right hemispheric fronto-basal ganglia network, including the right inferior frontal gyrus (rIFG), the pre-supplementary motor area (pre-SMA), and the basal ganglia (Aron and Poldrack, 2006; Aron et al., 2016; Hannah and Aron, 2021). The importance of the rIFG and pre-SMA in response inhibition is well supported by investigations of traumatic brain injury and transcranial magnetic stimulation (TMS) (Aron et al., 2003; Chambers et al., 2006; Floden and Stuss, 2006). In summary, the rIFG and pre-SMA are two critical brain regions for the effective execution of response inhibition, and methods aimed at simultaneously promoting the activity of these brain regions provide a new direction for improving response inhibition and treating patients with impaired response inhibition ability.

Transcranial direct current stimulation (tDCS) is a promising method to regulate cortical activity and enhance cognitive ability. Although there are some impact factors limiting the reliability of causal relationship revealed by tDCS, such as limited spatial precision and unwanted brain area activation, tDCS is still a good way to provide causal evidence for the links between brain function and corresponding behavioral changes (Filmer et al., 2014; Gbadeyan et al., 2016; Yavari et al., 2018). tDCS is non-invasive, safe, tolerable, and easy to operate (Bikson et al., 2016; Valiengo et al., 2020; Weidler et al., 2020). It transmits a weak direct current through electrodes placed on the scalp and influences the activity of the cerebral cortex (Nitsche and Paulus, 2000). Generally, anodal stimulation will increase the excitability of the cortex via subthreshold depolarization and long-term potentiation (LTP)-like plasticity, while cathodal stimulation decreases excitability via hyperpolarization and long-term depression (LTD)-like plasticity (Nitsche and Paulus, 2000, 2001; Pisoni et al., 2018).

Currently, tDCS has been widely used in studies on response inhibition, but the results are heterogeneous. Prior studies have revealed elevated response inhibition after anodal stimulation on the rIFG (Jacobson et al., 2011; Stramaccia et al., 2015; Li et al., 2019) and pre-SMA (Hsu et al., 2011; Kwon and Kwon, 2013a,b; Yu et al., 2015) in healthy young participants, indicating that the rIFG and pre-SMA are important targets for enhancing response inhibition using tDCS. However, contradictory results have also been reported, claiming that single-target tDCS over rIFG or pre-SMA is ineffective (Dambacher et al., 2015; Bender et al., 2017; Thunberg et al., 2020). Additionally, the majority of the tDCS studies targeting rIFG or pre-SMA in healthy participants employed conventional tDCS; few studies used high-definition tDCS (HD-tDCS) (Hogeveen et al., 2016). HD-tDCS, an optimized form of conventional tDCS, can produce more prominent behavioral and neurophysiological effects with more superior spatial precision compared with conventional tDCS, supporting its more widespread application (Kuo et al., 2013; Sehatpour et al., 2021). Taken together, this underscores the need to develop more potent protocols using HD-tDCS to improve response inhibition and clarify the validity of single-target tDCS.

It is well known that the normally effective execution of brain function is based on neural networks rather than on isolated brain regions (Ester and Kullmann, 2021). Simultaneous HD-tDCS with identical polarity on multiple functionally related brain regions – in other words, multitarget stimulation – can regulate cortical excitability more efficiently and enhance tDCS effects more prominently than single-target stimulation (Fischer et al., 2017; Hill et al., 2018; Ester and Kullmann, 2021; Gregoret et al., 2021). Multitarget HD-tDCS has been applied to studies of motor ability and working memory and the results have demonstrated that multitarget stimulation is more effective (Dagan et al., 2018; Hill et al., 2018). However, currently, studies of multitarget HD-tDCS for response inhibition have not been carried out. Therefore, this study aims to investigate the effects of multitarget HD-tDCS on enhancing response inhibition and compare them with the effects of single-target HD-tDCS.

To better understand the neural mechanism of tDCS-induced behavioral changes in response inhibition, relevant neurophysiological tools such as fMRI, positron emission tomography (PET), electroencephalography (EEG), and functional near-infrared spectroscopy (fNIRS) should be used in conjunction with behavioral tasks. However, the application of fMRI and PET is limited by their large bulk and immobility, and the accuracy of EEG signal is easily disturbed with the artifacts, so fNIRS may be the ideal tool for tDCS research (Yaqub et al., 2018; Yang et al., 2019; Friehs et al., 2021b), and it has been used to monitor hemodynamic changes induced by tDCS intervention (Yaqub et al., 2018; Lu et al., 2020). fNIRS is an optical and non-invasive neuroimaging method, with the advantages of greater tolerance to motion artifacts, high adaptability, portability, low cost, and participant-friendliness. Hence, fNIRS can overcome some limitations of the aforementioned imaging technologies. It can measure the concentrations of oxyhemoglobin and deoxyhemoglobin in brain tissue in a more natural situation (Scholkmann et al., 2014; Pinti et al., 2020; Veit et al., 2021). In recent years, fNIRS has been employed in the study of response inhibition and the results have revealed increased oxyhemoglobin concentrations in the prefrontal cortex during response inhibition (Herrmann et al., 2005; Hudak et al., 2017). However, few response inhibition studies have applied fNIRS to measure the neural activity of relevant brain regions before and after tDCS.

In order to overcome the limitations of previous studies, this study was designed to examine the effects of multitarget anodal HD-tDCS on improving response inhibition and to determine whether anodal stimulation of the rIFG or pre-SMA actually enhances response inhibition compared to sham stimulation. We hypothesized that HD-tDCS applied to rIFG + pre-SMA, rIFG, or pre-SMA could all enhance response inhibition compared with sham stimulation. We further anticipated that multitarget HD-tDCS could be more effective at improving response inhibition. As far as we know, this is the first study to examine the effect of multitarget anodal HD-tDCS on response inhibition and to compare the effects of different stimulation montages.

## Materials and methods

### Participants

A total of 92 healthy college students (mean age = 20.58 ± 1.54 years, range = 18 – 24 years, 43 males) participated in the experiment and were randomly divided into four groups: (1) multitarget anodal HD-tDCS (rIFG + pre-SMA condition), *n* = 22; (2) anodal HD-tDCS on the rIFG (rIFG condition), *n* = 24; (3) anodal HD-tDCS on the pre-SMA (pre-SMA condition), *n* = 22; and (4) sham stimulation, *n* = 24. All participants had normal or corrected-to-normal vision and were right-handed as assessed with the Edinburgh Handedness Inventory (Oldfield, 1971). Inattention and impulsivity were assessed by the Adult ADHD Self-report Scale (ASRS); only participants with an average score of 17 and below were included, as individuals with a sum score on either subscale of 17 or higher were considered likely to have ADHD (Kessler et al., 2005; Yeh et al., 2008). All participants were naive to the nature of the study and were screened to ensure that the final sample included only neurologically and psychiatrically healthy individuals without any contraindications (e.g., metal implants in the head, pregnancy, a history of seizures, etc.) to tDCS, and none of the participants reported taking any psychotropic medication. G*Power 3.1.9.6 was used to compute *a priori* sample size, and a minimum sample N of 48 (12 per group) was needed with a medium effect size of *f* = 0.25, a power of 1 - β = 0.80, and an α-value of 0.05 (Cohen, 1992; Faul et al., 2007). The groups were matched in basic characteristics (Table 1). Participants gave their written informed consent before the experiment. The study was approved by the Tangdu Hospital Ethics Committee and abided by the Declaration of Helsinki.

**TABLE 1:** Basic characteristics of participants (numbers or means and standard deviations).

Variable	rIFG + pre-SMA	rIFG	pre-SMA	sham	F/χ^2^	*p*
*n*	22	24	22	24		
Gender (male/female)^a^	11/11	11/13	10/12	11/13	0.124	0.989
Age (years)^b^	20.82 (1.47)	20.38 (1.66)	20.41 (1.76)	20.71 (1.27)	0.457	0.713
Education (years)^b^	15.68 (1.17)	15.29 (1.71)	15.41 (1.59)	15.75 (1.54)	0.483	0.695
ASRS-inattention^b^	11.41 (3.67)	9.46 (3.78)	11.64 (3.86)	10.63 (2.78)	1.790	0.155
ASRS-hyperactivity/impulsivity^b^	7.95 (4.13)	7.58 (4.03)	8.00 (3.95)	8.04 (4.29)	0.063	0.979

ASRS, Adult ADHD Self-report Scale; rIFG, right inferior frontal gyrus; pre-SMA, pre-supplementary motor area; a, χ^2^ test; b, one-way analysis of variance; p < 0.05 was considered significant.

### Design and procedure

**FIGURE 1:**
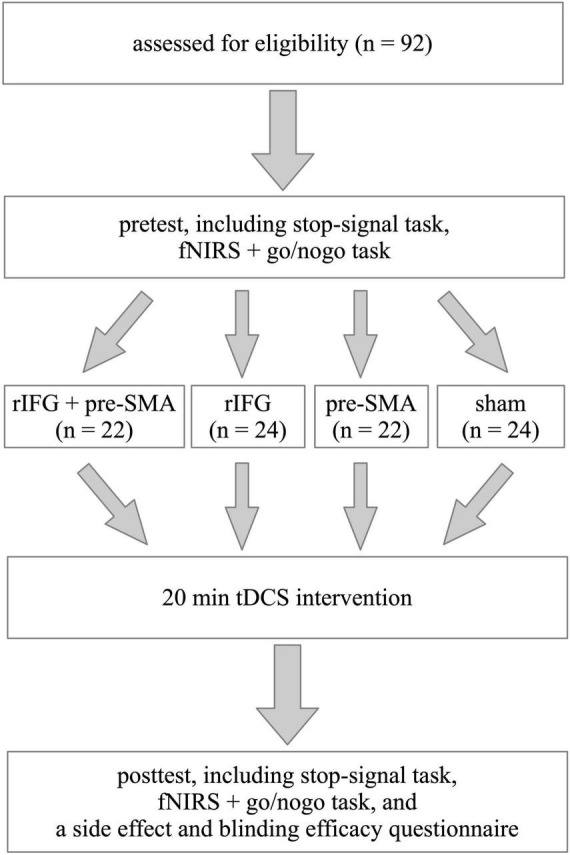
Schematic illustration of the study design. The experiment followed a single-blinded, randomized, between-subject, sham-controlled, and pretest-posttest design.

The experiment followed a single-blind, randomized, between-subject, and sham-controlled design (Figure 1). The two important tasks used to study response inhibition are the stop-signal task (SST) and the go/nogo task (GNG) (Cunillera et al., 2016). In this study, we employed both SST and GNG in order to increase the robustness of the results. To detect neural changes, we chose to collect fNIRS data during behavioral tasks. However, we only recorded fNIRS signals during GNG but not during SST because the design of fNIRS recording was drawing upon previous studies, which utilized SST and GNG as behavioral assessment but only recorded fNIRS during GNG (Hudak et al., 2017). Additionally, collecting fNIRS signals during both SST and GNG takes more time to record, which may make participants feel uncomfortable because they need to keep still in the process. Before the experiment, participants participated in a brief interview to collect basic demographic information, complete the ASRS, and screen for their eligibility for tDCS. Each participant completed a pretest including SST and GNG in a counterbalanced order and fNIRS data were recorded during the GNG. Then they were randomly assigned to receive one of the four types of single-session stimulation. After tDCS application, they immediately received a posttest identical to the pretest as well as a questionnaire to evaluate side effects and blinding efficacy. Tasks were programmed and run on E-prime 3.0 software (Psychology Software Tools, Inc., Sharpsburg, PA). Before starting each task, participants were given instructions on how to complete it. The whole experiment was performed within 120 min.

### High-definition transcranial direct current stimulation

**FIGURE 2:**
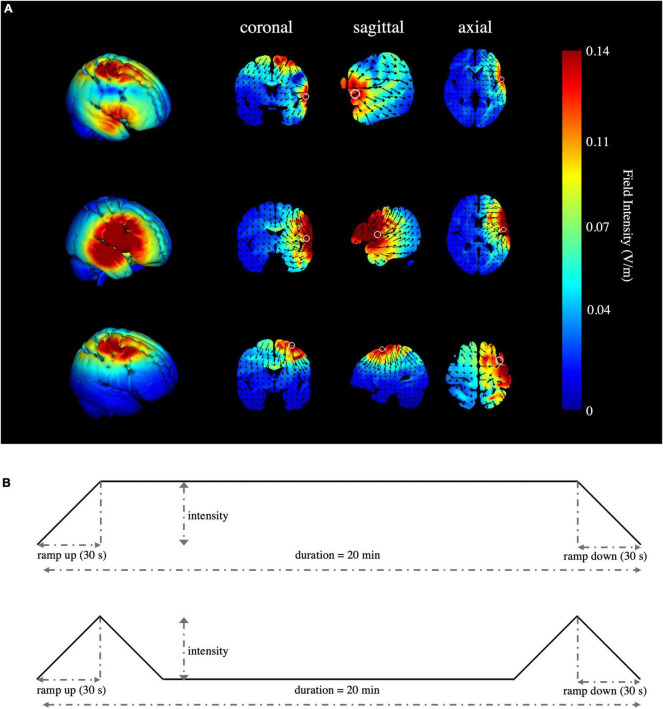
Simulated stimulation conditions and time progression of tDCS. **(A)** Simulated electric field and current flow of rIFG + pre-SMA (top), rIFG (middle), and pre-SMA (bottom). The color bar represents the field intensity and the arrow points in the direction of current flow. Column 1 is a 3D view, while column 2 to column 4 range from coronal to axial slices. **(B)** Schematic illustration of the duration of tDCS, ramp-up, and ramp-down periods for verum (top) and sham stimulation (bottom). The current intensity was delivered at 1.25 mA for single-target HD-tDCS, 2.5 mA for multitarget HD-tDCS, and 1.25 mA or 2.5 mA in a pseudo-random order for the sham stimulation condition.

High-definition transcranial direct current stimulation (HD-tDCS) was applied by a Soterix Medical MXN-9 High-Definition Transcranial Electrical Stimulator (Soterix Medical, Inc., New York, United States). This study followed all procedures for using HD-tDCS as demonstrated previously (Villamar et al., 2013). The electrodes were localized using the 10-10 EEG system (Jurcak et al., 2007). The montage was determined and the corresponding electric field and current flow were generated (Figure 2A) using HD-Targets and HD-explore software (Soterix Medical, Inc., New York, United States). This method has been widely used in prior studies and has proven to be effective (Nikolin et al., 2015; Hogeveen et al., 2016; Reinhart and Nguyen, 2019; Maldonado and Bernard, 2021). The parameters of each electrode in each verum stimulation condition are listed below (Table 2). Participants in the sham stimulation condition were pseudo-randomized to receive one of the three verum stimulation montages (Hill et al., 2018). The pseudo-randomization is different from randomization because it is generated by some algorithms. In this study, participants in the sham stimulation condition were sorted according to the ascending order of their names and were labeled with number 1, 2, and 3 in order. Number 1, 2, and 3 represented the participant received the rIFG + pre-SMA condition, rIFG condition, and pre-SMA condition, respectively. All conditions were conducted with the same electrode placement as the multitarget condition with only the currents changed for blinding purposes (Schneider et al., 2021; Zhou et al., 2021). The panel of the instrument was not visible to the participants. HD-tDCS was delivered at 2.5 mA for multitarget stimulation and 1.25 mA for single-target stimulation. These intensities have been proven safe and reliable enough to improve cognitive performance (Villamar et al., 2013; Hogeveen et al., 2016; Abellaneda-Perez et al., 2021; Zhou et al., 2021). Verum stimulation was applied for 20 min with a ramp up and ramp down of 30 s each. Sham stimulation consisted of a 30 s ramp up and a 30 s ramp down at the beginning and the end, respectively, with no current during the intervening time (Figure 2B), facilitating blinding by mimicking the sensations of verum tDCS without actual neurological changes (Di Rosa et al., 2019; Sharma et al., 2021). After stimulation, participants were asked whether they received verum or sham stimulation and how confident they were based on a scale from 0 (complete guess) to 10 (absolutely sure), Additionally, another 11-point scale was used to evaluate the intensity of any sensations (e.g., itching, tingling, metallic taste, or burning) they felt during the stimulation, with 0 = no sensation and 10 = strongest sensation imaginable (Hill et al., 2017).

**TABLE 2:** Location and current intensity (mA) of each electrode for each verum stimulation condition, according to the international 10-10 system.

Electrode location	rIFG + pre-SMA	rIFG	pre-SMA
Fz	–0.51	0.00	–0.32
C2	1.48	0.00	1.25
FC4	–0.41	–0.32	–0.31
C4	–0.52	–0.31	–0.31
P4	–0.36	0.00	–0.31
FT8	1.02	1.25	0.00
FT10	–0.53	–0.31	0.00
TP8	–0.17	–0.31	0.00
Total current	2.50	1.25	1.25

### Stop-signal task

**FIGURE 3:**
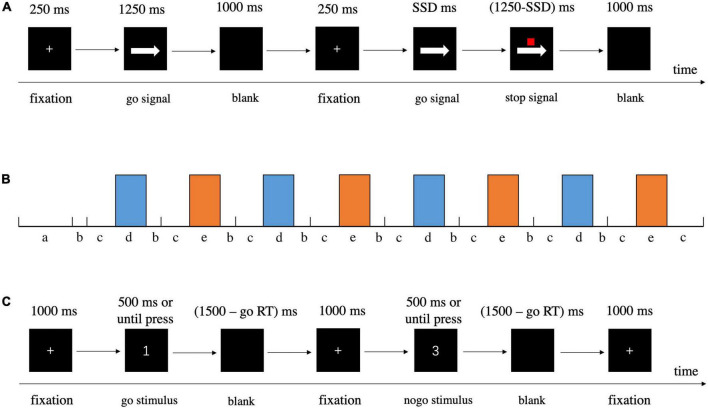
Detailed information about procedures of behavioral tasks. **(A)** SST. **(B)** Schematic illustration of block design for GNG, a = instruction, b = cue, c = rest, d = go block, e = nogo block. **(C)** GNG.

We employed a valid and reliable behavioral task, the stop-signal task (SST), to investigate response inhibition (Logan et al., 1984; Verbruggen and Logan, 2008; Verbruggen et al., 2019), in which participants responded to a go stimulus (also referred to as the primary task). Occasionally, the go stimulus was unpredictably followed by a stop signal at irregular intervals; the stop signal instructed participants to withhold their response. The SST settings we applied were consistent with the current consensus (Verbruggen et al., 2019). On the prepotent go stimuli (75% of total trials), participants were required to press “F” on the keyboard with their right index finger in response to left arrows and press “J” with their right ring finger in response to right arrows as quickly and accurately as possible. However, on a minority of trials (25%), a small red square (stop signal) was presented above the arrow after an interval (stop signal delay, SSD), indicating the need to cancel the planned response. The SSD started at 250 ms and was dynamically adjusted by a tracking procedure (50 ms increment/decrement for successful stopping/unsuccessful stopping, range = 0 – 1250 ms) to ensure that each participant successfully inhibited about 50% of the stop trials. Details about the task procedures and the duration of fixation, stimulus presentation, and blank are displayed in Figure 3A. Besides a practice block of 48 trials (25% stop-signal trials), there were 200 trials in the test block, including 150 go trials and 50 stop-signal trials, all presented in a randomized order. We estimated the covert latency of the inhibition process by using the stop-signal reaction time (SSRT) as calculated by the mean method, which subtracted the mean SSD from the mean reaction time in all correct go trials when the overall stop accuracy converged at 0.5 (Logan et al., 1984; Verbruggen and Logan, 2009; Hogeveen et al., 2016; Bartholdy et al., 2019; Chen et al., 2020), with shorter SSRTs indicating superior response inhibition. In addition to SSRT, stop accuracy (the probability of correctly withholding responses on stop trials) and goRT (mean RT on correct go trials) were also assessed.

### Go/nogo task

The go/nogo task was designed to induce response inhibition. In the current study, we recorded fNIRS during GNG, and thus we redesigned the task in a block-design paradigm (Figure 3B). The task began with a go block with subsequent blocks alternating between go and nogo (four repetitions each, and 12 trials per block) separated by rest blocks, with each block lasting 30 s (Herrmann et al., 2005; Hudak et al., 2017). Prior to the actual fNIRS measurement, participants were given instructions for the following two task blocks, and they were told to sit in a relaxed position and keep still to avoid head movements. A 5 s cue appeared before each rest block to alert participants whether the next block was a go or nogo block (Nagashima et al., 2014; Lu et al., 2020). In the go block trials, participants were asked to respond as quickly and accurately as possible to each stimulus (number 1, 2, and 4) by pressing “J” on the keyboard with their right index finger. In the nogo block trials, the participants had to press “J” as quickly and accurately as possible in response to the go stimuli (number 1, 2, and 4), whereas they were instructed to withhold their response to the nogo stimulus (number 3) following the fixation cross. For the go blocks, all 12 trials were go stimuli, and for nogo blocks, both go and nogo stimuli had the same occurrence probability of 0.5. In total, the go/nogo task maintained a ratio of 75% go and 25% nogo trials (Herrmann et al., 2005; Nagashima et al., 2014; Rodrigo et al., 2014). For each trial, a fixation cross appeared in the center of the screen for 1000 ms, and then the number stimulus was presented for a maximum of 500 ms or until reaction. Once the participant responded to the stimulus, it disappeared immediately and a blank screen appeared. The stimulus and blank were together presented for 1500 ms (Figure 3C) (Herrmann et al., 2005). In addition to goRT and nogo accuracy (the possibility of successful inhibition in nogo trials), inverse efficiency score (IES) was analyzed and adopted as the primary outcome. IES may be a better indicator to measure GNG performance in consideration of the tradeoff between speed and accuracy, with a lower value reflecting higher performance (Bruyer and Brysbaert, 2011; Zhao et al., 2018). IES was calculated by dividing goRT by the percentage of all correct responses (the number of correct go trials and nogo trials divided by the total number of trials).

### Functional near-infrared spectroscopy

**FIGURE 4:**
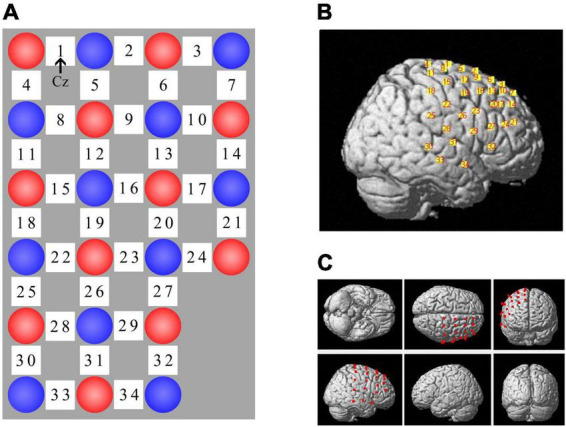
fNIRS channel layout. **(A)** Optode arrangement, red circle = source, blue circle = detector, white square = channel. Channel 1 was located at Cz and the uppermost edge (i.e., channel 1 – 3) of the probe set overlapped with Cz-Oz. **(B)** Spatial registration of channels on a rendered brain. **(C)** Different views of optode locations.

Changes in oxygenated (oxy-Hb) and deoxygenated (deoxy-Hb) hemoglobin were measured using the LABNIRS fNIRS system (Shimadzu Co., Kyoto, Japan) during GNG. We used 11 sources (emitting light at 780 nm, 805 nm, and 830 nm) and 11 detectors to form 34 measurement channels over the right cerebral cortex, including the regions of interest (i.e., the pre-SMA and rIFG), with a raw sample rate of 27.78 Hz. Participants were fitted with a headcap with optode holders to set the source-detector distance at 3 cm. For consistency of optode placement across participants, channel 1 was located at the Cz point of the international 10–20 EEG system (Jasper, 1958) and the uppermost edge of the probe set overlapped with Cz-Oz (Figure 4A). To determine the anatomical locations of optodes and channels, we used a digitizer (Fastrak, Polhemus, Colchester, VT, United States) to capture the 3D coordinates of optode positions based on head landmarks (nasion, Cz, and left and right preauricular points) in real-world space and registered fNIRS coordinates of channels and optodes on the standard Montreal Neurological Institute (MNI) template using the software package NIRS-SPM (Figures 4B,C) (Ye et al., 2009; Orcioli-Silva et al., 2021). Finally, we estimated the corresponding relationship between fNIRS channels and the anatomical structural labels in the Brodmann areas and LPBA40 according to the channels’ coordinates (Tsuzuki et al., 2007; Ye et al., 2009; Nagashima et al., 2014). We stipulated in advance that if the percentage of overlap exceeded 50%, the channel represented the corresponding brain area. Finally, each region of interest (ROI) consisted of corresponding channels. Nine channels labeled the pre-motor and supplementary motor cortex in the Brodmann areas (channel 1/2/5/6/8/9/12/19/26) and 2 channels represented the right inferior frontal gyrus using LPBA40 (channel 24/27).

### Data preprocessing

We preprocessed raw fNIRS data in the Homer2 fNIRS analysis package (Huppert et al., 2009) based on Matlab R2013b software. At first, raw data was down-sampled to 9.26 Hz after being imported into Homer2 and was visually inspected to ensure there was no totally bad signal channel. Next, optical intensity was converted to optical density (OD) by the hmrIntensity2OD function. Motion artifacts were identified by a hmrMotionArtifactByChannel function and corrected by a spline interpolation method in every participant (Scholkmann et al., 2010; Zhang et al., 2021). Then the fNIRS signals were bandpass-filtered with cutoff frequencies of 0.01 and 0.1 Hz to eliminate physiological noise (e.g., heartbeat and respiration) and correct drift artifacts throughout the experimental process. According to the modified Beer-Lambert law, the filtered OD signal was transformed to relative concentration signal data for oxy-Hb, deoxy-Hb, and total-Hb. Finally, we used a 5-s period prior to the onset of each block as a baseline to standardize hemodynamic changes during the 30 s task block and calculated a block-averaged relative concentration change for the two conditions (go and nogo) over the time range. We decided to analyze only oxy-Hb data because oxy-Hb is more reliable and sensitive to brain activity changes relative to deoxy-Hb or total-Hb (Hoshi et al., 2001; Nagashima et al., 2014; Ehlis et al., 2016; Lu et al., 2020; Zhuo et al., 2022). But we also provided the relevant deoxy-Hb and total-Hb data in the Supplementary Material.

With regard to the behavioral data, 11 participants were excluded from further analysis in the SST because they showed (1) stop accuracy <0.25 or >0.75 (Congdon et al., 2012), which might result from participants’ strategic behavior and not complying with the task instruction, such as waiting for the stop signal to show (obtaining a high stop accuracy) and pressing the key too fast throughout the task (obtaining a low stop accuracy); (2) goRT > 1000 ms (Enge et al., 2014) or (3) violation of the independent race model, implying the mean RT on unsuccessful stop trials is greater than goRT (Verbruggen and Logan, 2009). After exclusion, the SST analysis was based on *n* = 20 for the multitarget condition, *n* = 22 for the rIFG condition, *n* = 20 for the pre-SMA condition, and *n* = 19 for the sham stimulation group. One participant was excluded from the GNG and fNIRS analyses due to an error rate exceeding 40% (Enge et al., 2014). Therefore, the final sample for the GNG and fNIRS data analyses included 91 participants (*n* = 22, 24, 22, and 23 for groups 1–4, respectively).

### Statistical analyses

IBM SPSS (version 26.0) software was used to conduct statistical analyses. The fNIRS data were analyzed by creating oxy-Hb contrasts for the nogo block minus the go block (△oxy-Hb = oxy-Hb_nogo_ – oxy-Hb_go_) (Veit et al., 2021). △oxy-Hb signals from channel 1/2/5/6/8/9/12/19/26 were averaged to represent pre-SMA activity, and those from channel 24/27 were averaged to yield rIFG activity. The data of △deoxy-Hb and △total-Hb are provided in the Supplementary Material. Categorical variables were examined by the chi-square test. One-way analysis of variance (ANOVA) was used to test data measured once and baseline performance, and the Kruskal–Wallis test was used for data with a skewed distribution. Whether the tracking procedure in SST obtained a stop accuracy of approximately 0.5 was verified using one-sample *t*-tests. The effects of tDCS stimulation were assessed with repeated measures ANOVA (RM-ANOVA) with time (pretest and posttest) as the within-subject factor and stimulation condition (multitarget, rIFG, pre-SMA, and sham stimulation) as the between-subject factor. *Post hoc* analyses were performed using Bonferroni-corrected pairwise comparisons. Considering the limitation of the classic frequentist approach, we used Bayesian analysis to further investigate the non-significant interaction effect in order to strengthen the robustness of our results. This was performed using JASP software (version 0.14.1.0) with a default Cauchy prior distribution with γ = 0.707. The Bayes factor BF_10_ represented the ratio of the possibility that the data favored the alternative hypothesis (H_1_) compared to the null hypothesis (H_0_). A BF_10_ superior to 3 indicated at least moderate evidence for H_1_. A BF_10_ between 1/3 and 3 indicated anecdotal evidence for H_0_ and H_1_, while a BF_10_ score between 1/10 and 1/3 represented moderate evidence for H_0_ and inferior to 1/10 signified strong evidence for H_0_ (Wagenmakers et al., 2011, 2018a,b). Further analysis explored the effect of each tDCS condition on the SSRT. Participants were allocated to the high-performance (HP) and low-performance (LP) subgroups in each condition by a median split method based on baseline SSRT (Whelan et al., 2012). An independent samples *t*-test was employed to compare the SSRT of the HP and LP subgroups in each condition. Further analysis was performed using a 2 (time: pretest and posttest) × 2 (subgroup: HP and LP) RM-ANOVA. In all analyses, *p* < 0.05 was considered statistically significant. In addition, for ANOVAs, effect sizes were reported as partial eta-squared (η^2^_p_).

## Results

### Behavioral data

#### Baseline

As shown in Table 3, the one-way ANOVAs revealed no significant difference (*p*s > 0.05) in any of the indices for SST and GNG between the four groups before tDCS intervention, thereby ensuring that any performance changes between the pretest and the posttest would be attributable to the tDCS stimulation.

**TABLE 3:** Means and standard deviations of behavioral task performance at baseline.

Task	rIFG + pre-SMA	rIFG	pre-SMA	sham	F	*p*
Stop-signal task						
SSRT	291.31(33.43)	267.11(33.65)	274.34(31.87)	277.12(29.9)	2.036	0.116
stop accuracy	0.51(0.06)	0.51(0.04)	0.50(0.05)	0.53(0.06)	0.936	0.428
goRT	543.19(185.40)	515.47(159.84)	497.63(156.17)	591.42(212.05)	1.023	0.387
Go/nogo task						
IES	388.42(67.52)	365.88(66.65)	378.46(59.73)	380.91(67.34)	0.476	0.700
goRT	371.41(56.07)	352.07(55.38)	364.58(56.22)	359.23(45.52)	0.539	0.657
nogo accuracy	0.93(0.07)	0.92(0.06)	0.92(0.06)	0.90(0.09)	1.028	0.384

goRT, mean reaction time on correct go trials; IES, inverse efficiency score; SSRT, stop-signal reaction time; rIFG, right inferior frontal gyrus; pre-SMA, pre-supplementary motor area. Besides the accuracy indicators, the units of the other measurements were milliseconds (ms). p < 0.05 was considered significant.

#### Stop-signal task

**FIGURE 5:**
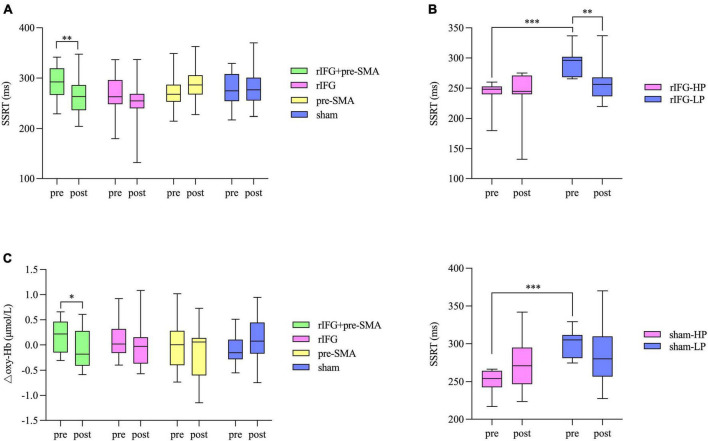
Box and whisker plots, showing the effects of HD-tDCS on the outcome measures. **(A)** Behavioral performance in SST. **(B)** Significant interaction effects between subgroup and time for the rIFG condition (top) and sham condition (bottom) in the further analysis for SST. HP, high-performance subgroup; LP, low-performance subgroup. **(C)** Changes in △oxy-Hb from pretest to posttest in the pre-SMA ROI. Boxes extend from the 25 to 75th percentiles with a horizontal line representing the median. Whiskers show the min to max values. *p < 0.05, ^**^p < 0.01, and ^***^p < 0.001.

One-sample *t*-tests indicated there was no significant difference between stop accuracy and 0.5 either in pretest or posttest for group (*p*s > 0.05). The number “0.5” refers to the optimal stop accuracy ensured by the aforementioned tracking procedure. RM-ANOVA showed a significant interaction effect between time and stimulation condition (*F*_(3,77)_ = 4.196, *p* = 0.008, η^2^_p_ = 0.141) for SSRT. *Post hoc* analysis revealed a significant decrease in SSRT after multitarget tDCS (*p* = 0.005). However, no significant difference was found after rIFG (*p* = 0.057), pre-SMA (*p* = 0.109), or sham stimulation (*p* = 0.717) (Figure 5A). The main effects were not significant (*p*s > 0.05). The baseline SSRT was significantly shorter in the HP subgroup relative to the LP subgroup for each condition after independent samples *t*-tests (*p*s < 0.001). Further analysis revealed that the main effect of time (*F*_(1_,_18)_ = 7.547, *p* = 0.013, η^2^_p_ = 0.295) was significant in the multitarget condition, manifesting significant smaller SSRT after stimulation (mean = 267.39 ms, SD = 37.81 ms) compared with pre-stimulation (mean = 291.31 ms, SD = 33.43 ms) regardless of subgroup. For the rIFG condition, the interaction effect between time and subgroup (*F*_(1_,_20)_ = 4.56, *p* = 0.045, η^2^_p_ = 0.186) and the effect of subgroup (*F*_(1_,_20)_ = 4.56, *p* = 0.003, η^2^_p_ = 0.357) were significant. *Post hoc* analysis showed only the LP subgroup experienced reduced SSRT (*p* = 0.009) (Figure 5B). Only the subgroup effect was significant in the pre-SMA condition (*F*_(1_,_18)_ = 26.429, *p* < 0.001, η^2^_p_ = 0.595), which indicated that no subgroups significantly changed SSRT after stimulation. For sham stimulation, the interaction effect was significant (*F*_(1_,_17)_ = 4.877, *p* = 0.041, η^2^_p_ = 0.223), but *post hoc* tests showed no significant difference in SSRT between pretest and posttest for both subgroups (*p*s > 0.05) (Figure 5B). There were no significant interaction effects for stop accuracy or goRT (*p*s > 0.05), and none of the main effects reached significance (*p*s > 0.05). Bayesian analysis showed moderate evidence for the null hypothesis that there was no interaction effect for stop accuracy (BF_10_ = 0.13) or for goRT (BF_10_ = 0.13).

#### Go/nogo task

The main effect of time was significant for IES due to a pre-to-post decrease for all conditions (*F*_(1_,_87)_ = 14.948, *p* < 0.001, η^2^_p_ = 0.147), but the main effect of condition and the interaction effect were not significant (*p*s > 0.05). Bayesian analysis showed a BF_10_ of 0.07 for the IES interaction term, indicating strong evidence for the null hypothesis. RM-ANOVA revealed a significant time effect for goRT driven by a decrease in RT after intervention under all conditions (*F*_(1_,_87)_ = 27.645, *p* < 0.001, η^2^_p_ = 0.241). There was no main effect of condition and no interaction effect for goRT (*p*s > 0.05). Bayesian analysis revealed moderate evidence to support the absence of an interaction term (BF_10_ = 0.11). None of the main effects or interaction effect reached significance for nogo accuracy (*p*s > 0.05). Strong evidence in favor of no interaction effect was established by BF_10_ = 0.09.

### Functional near-infrared spectroscopy data

The baselines of △oxy-Hb were matched between the four tDCS conditions in both the pre-SMA (*F*_(3_,_87)_ = 2.546, *p* = 0.061, η^2^_p_ = 0.081) and rIFG (*F*_(3_,_87)_ = 0.274, *p* = 0.844, η^2^_p_ = 0.009). In the pre-SMA region, an interaction effect between time and stimulation condition was found (F_(3_,_87)_ = 3.023, *p* = 0.034, η^2^_p_ = 0.094), and *post hoc* analysis indicated that △oxy-Hb significantly decreased after the multitarget stimulation (*p* = 0.026). Although no significant differences in the other three groups were detected (*p*s > 0.05), a pre-to-post decrease in △oxy-Hb was observed under the rIFG and pre-SMA conditions but not in the sham stimulation condition (Figure 5C). The main effects of time and condition did not reach significance (*p*s > 0.05). In the rIFG region, RM-ANOVA revealed no significant main effects or an interaction effect for △oxy-Hb (*p*s > 0.05) (Supplementary Figure 1). Bayesian analysis revealed strong evidence supporting the null hypothesis that there was no interaction effect (BF_10_ = 0.08).

### Side effects, blinding efficacy, and electric field modeling

All participants tolerated the tDCS procedure well and there were no serious side effects reported. The ratings of the intensity of sensations between groups were similar (*F*_(3_,_88)_ = 0.521, *p* = 0.669, η^2^_p_ = 0.017). There were 21, 23, 19, and 22 participants in conditions one to four respectively who reported that they received real stimulation. No significant difference (χ^2^ = 1.764, *p* = 0.656) was found in the number of participants speculating whether they received real or sham stimulation, and the confidence scores were insignificant according to the Kruskal–Wallis test (*p* = 0.589). The electric field simulation confirmed the focal electric field over the pre-SMA and rIFG for the multitarget condition, rIFG for the rIFG condition, and pre-SMA for the pre-SMA condition. The rIFG stimulation condition produced much higher electric field intensity in the targeted cortex than either the multitarget or pre-SMA conditions (Figure 2A).

## Discussion

Response inhibition is a critical part of executive function, and thus it is worthwhile to investigate how response inhibition ability can be more effectively improved through tDCS. The present study aimed to investigate the effect of different stimulation montages on response inhibition as assessed by behavioral and neuroimaging methods. Behavioral data showed a significant decrease in SSRT after multitarget HD-tDCS that did not exist in the other conditions. Further analysis showed that significant reductions in SSRT were present in both the LP and HP subgroups after multitarget stimulation, as well as in the LP subgroup after rIFG stimulation. fNIRS data showed that only multitarget stimulation produced a significantly lower △oxy-Hb in the pre-SMA. However, pre-SMA tDCS modulated neither SSRT nor fNIRS signals significantly from pretest to posttest. The other indices in SST or GNG were not substantially altered for the real stimulation conditions relative to the sham stimulation condition. All null hypotheses of interaction effects between time and stimulation condition were confirmed by Bayesian analysis.

To the best of our knowledge, this is the first study to provide evidence that multitarget stimulation is an effective way to improve response inhibition and is more potent than the commonly used single-target HD-tDCS. SSRT decreased significantly after rIFG + pre-SMA stimulation, indicating improved response inhibition. However, the pre-to-post changes in SSRT did not reach significance under the other three conditions. It has been reported that tDCS effects are dependent on initial performance, with greater tDCS effects observed in those with poor baseline performance; better baseline performance is related to higher neural excitability, which is difficult to elevate further (Wu et al., 2021a,b). The HP subgroup and the LP subgroup were divided using a median-split method in the present study (Whelan et al., 2012). After statistical comparisons, the results showed the HP subgroup had a significantly shorter SSRT relative to LP subgroup for each condition. Further analysis revealed that multitarget tDCS improved SSRT in both the HP subgroup and the LP subgroup compared with the rIFG condition which improved SSRT only in the LP subgroup. Coupled with the fact that none of the changes were seen in either subgroup for the pre-SMA tDCS condition, the results indicate that multitarget HD-tDCS yielded the most pronounced effects of all the conditions tried. This result is consistent with published studies indicating that multitarget stimulation produces larger effects relative to single-target tDCS (Vaseghi et al., 2015; Dagan et al., 2018). Additionally, the electric field modeling results showed the rIFG condition yielded greater electric field intensity in the targeted cortex compared with the multitarget condition and pre-SMA condition. However, the measurement results illustrated the multitarget stimulation is more beneficial to improving response inhibition. The results seemed contradictory because of the assumption that electric field intensity in a brain area directly associates with the behavioral effect of tDCS (Evans et al., 2020). One highly possible explanation may lie in that efficient execution of brain function is based on networks of brain areas rather than individual brain regions (Hoogman et al., 2017; Ester and Kullmann, 2021); and multitarget stimulation tries to modulate the associated brain network and may result in additive effects of tDCS on performance compared with single-target stimulation (Brem et al., 2018; Ester and Kullmann, 2021; Friehs et al., 2021a; Gregoret et al., 2021). Additionally, there is some evidence for the potentially inverted U-shaped nature of tDCS interactions with behavior performance, in which an intensity may lead to better performance when it lies closer to the peak of the inverted-U curve (Ehrhardt et al., 2021). According to the electric field modeling results, if the site of action of stimulation was just the IFG, the multitarget condition seemed to apply a much lower stimulation intensity to the IFG than the IFG stimulation condition; this stimulation intensity produced by multitarget condition may lie near the peak of the inverted-U curve. In this way, the inverted U-shaped intensity response curve may partly account for the difference between the rIFG condition and multitarget condition. Consequently, the present study verifies our hypothesis and provided preliminary evidence that multitarget tDCS is a more effective montage for enhancing response inhibition and fills a research gap on enhancing response inhibition using a multitarget montage. However, additional studies are warranted to confirm whether it is the particular case with our selected stimulation intensities.

We found that the rIFG condition was effective in improving response inhibition, although only for the low-performance participants. The favorable effect of rIFG stimulation relative to sham stimulation is consistent with previous results (Jacobson et al., 2011; Stramaccia et al., 2015; Li et al., 2019). However, single-target tDCS over the pre-SMA did not improve response inhibition. On the one hand, this result contradicts previous studies reporting significant reductions in SSRT after anodal tDCS over the pre-SMA (Kwon and Kwon, 2013a,b; Yu et al., 2015). One factor that might account for the discrepancy could be the different stimulation parameters employed in these studies (Mayer et al., 2020; Schneider et al., 2021). Prior studies used conventional tDCS with large pad electrodes ranging from 16 to 35 cm^2^ (Kwon and Kwon, 2013a,b; Yu et al., 2015), leading to low spatial resolution and distributed current. Consequently, it is highly possible that other brain regions related to response inhibition were stimulated in a complex way (Chen et al., 2021). However, the present study used HD-tDCS with small circular electrodes (1.2 cm diameter), and the center anode was surrounded by return electrodes, yielding greater spatial precision relative to conventional tDCS (Kuo et al., 2013; Sehatpour et al., 2021). Hence, HD-tDCS reduces the confounding impact of other brain regions relative to conventional tDCS, making the causal relationship between brain stimulation and relevant behavioral changes more convincing. In addition, the placement of electrodes might also lead to inconsistency because the anode electrode was placed over C2 in the present study, and the center of the pad electrode was put over Fz (Yu et al., 2015) or 4 cm anterior to Cz (Kwon and Kwon, 2013a,b) in previous studies. On the other hand, the absence of improvement in SSRT is consistent with the results of some other studies (Bender et al., 2017; Fujiyama et al., 2021), suggesting that more studies are needed to figure out the effect of anodal tDCS over the pre-SMA.

Corresponding cortical activity is critical for the execution of response inhibition and is directly related to cerebral blood flow. We measured hemodynamic responses in the rIFG and pre-SMA during a go/nogo task using fNIRS and found that △oxy-Hb was significantly reduced in the pre-SMA after multitarget stimulation compared to baseline. There was also a decrease of △oxy-Hb in both the rIFG and pre-SMA stimulation conditions, although it was not significant. This decrease could be considered as a biomarker of improved neural efficiency representing a more efficient neural network, defined as the quantity of performance-related changes accomplished by per neuron activity (Zarahn et al., 2007; Enriquez-Geppert et al., 2013). Neural efficiency has been discussed in prior studies, which found that behavioral performance was unchanged or improved even though the corresponding brain activity was decreased using tDCS (Holland et al., 2011; Lu et al., 2020; Orcioli-Silva et al., 2021). We found that HD-tDCS intervention might reduce the amount of energy the brain needs to finish the same GNG without changing performance. In particular, the multitarget HD-tDCS significantly decreased △oxy-Hb, demonstrating substantially improved neural efficiency, which is further proof of the advantage of using multitarget stimulation. However, in contradiction to prior studies that showed brain activity in the rIFG (Herrmann et al., 2005; Rodrigo et al., 2014), we did not observe a significant change of △oxy-Hb in that region even when using multitarget HD-tDCS. One possible explanation is that the probe set covering the rIFG was located at the border of the rIFG, so there were only two channels representing the rIFG. Therefore, the fNIRS in our study may have failed to measure the activity of the rIFG reliably and reflect the true changes. Besides, the channels representing pre-SMA in our study included relatively lateral channels (e.g., channels 19 and 26), meaning the pre-SMA may not be specific enough. This may occur because there only exists the “Pre-Motor and Supplementary Motor Cortex” anatomical label in the standard brain template when we anatomically labeled fNIRS channels, which limits us to further segment the brain region. We had to regard this label as the pre-SMA label. This practice is in line with previous studies which also used the pre-SMA to refer to the “Pre-Motor and Supplementary Motor Cortex” (Wang et al., 2021).

Although the present study provides preliminary evidence for the advantages of multitarget tDCS for improving response inhibition, some limitations should be considered. First, the participants were all young healthy adults in our study; consequently, the results should be cautiously generalized to other groups with different ages. Considering neural anatomical differences, the effect of tDCS over the rIFG and pre-SMA on response inhibition has been shown to be age-dependent (Fujiyama et al., 2021), so future studies are warranted to further elucidate age-related differences in the results of tDCS application. Second, there was no follow-up assessment and thus the sustainability of the effects of multitarget tDCS remains unclear; this is a vital issue for the use of tDCS in practical applications. Some studies have reported that a single session of conventional tDCS-induced (1 mA, 13 min) excitability changes could last for 90 min (Nitsche and Paulus, 2001) and single-target HD-tDCS (2 mA, 20 min) has shown a lasting after-effect for more than 2 h (Kuo et al., 2013). Hence, additional studies with follow-up measurements are needed to illuminate the duration of the after-effect of multitarget HD-tDCS. Third, the brain activity in the rIFG has yet to be clarified. For future work, more accurate probe placement should shed more light on the neurophysiological changes that take place after tDCS intervention. Fourth, the brain region of the pre-SMA needs to be more specific. It is recommended for future studies to utilize MRI to obtain more fine-grained segmentation of the brain region. Besides, the present study adopted a single-blind and between-group design, which might weaken the power of the results (Lu et al., 2020; Friehs et al., 2021b). Therefore, more rigorous experimental designs are recommended for future studies. Moreover, the electric field intensity differed in different stimulation conditions. Although it did not impact the interpretation of the findings in this study, future studies should carefully consider to normalize to produce roughly equivalent electric field intensity at the cortex. Finally, the multitarget HD-tDCS protocol in this study, including electrode positions and current intensity, was determined based on a generic head model rather than personalized adjustment. However, due to the inter-individual variability of cortical excitability changes in response to stimulation, the standardized “one size fits all” application of the multitarget HD-tDCS stimulation protocol may not be generalized well to other clinical individuals (Mizutani-Tiebel et al., 2022). Personalized application of multitarget stimulation protocol should be further explored in future studies.

## Conclusion

The present study has demonstrated that multitarget HD-tDCS improved response inhibition. Both high-performance and low-performance participants showed a significant reduction in SSRT after multitarget stimulation, whereas only the low-performance participants yielded a significantly decreased SSRT after the rIFG stimulation. We did not observe any significant improvements in SSRT after the pre-SMA stimulation and sham stimulation. Other indicators in behavioral tasks were not significantly altered for the verum stimulation conditions compared with the sham stimulation condition. fNIRS signals recorded during GNG showed a decrease in △oxy-Hb under all three verum tDCS conditions in the pre-SMA region, interpreted as sharpened neural efficiency, but the decrease reached statistical difference only for multitarget tDCS. This study thus provides preliminary evidence that multitarget HD-tDCS over the rIFG and pre-SMA is likely to be the most potent protocol for enhancing response inhibition ability in healthy individuals. It also lays a solid theoretical basis for clinical utility and provides new progress for the treatment of response inhibition deficits.

## Data availability statement

The original contributions presented in this study are included in the article/Supplementary material, further inquiries can be directed to the corresponding authors.

## Ethics statement

The studies involving human participants were reviewed and approved by the Ethics Committee of Tangdu Hospital. The patients/participants provided their written informed consent to participate in this study.

## Author contributions

ZG, YG, XZ, and XY conceived the study design. ZG, YG, HL, RQ, and XW performed the participants’ recruitment and data collection. ZG and YG performed data analysis. ZG wrote the draft of the manuscript. XZ obtained funding and contributed to the manuscript revision. All authors contributed to the article and approved the submitted version.

## References

[B1] Abellaneda-Perez K. Vaque-Alcazar L. Perellon-Alfonso R. Sole-Padulles C. Bargallo N. Salvador R. (2021). Multifocal transcranial direct current stimulation modulates resting-state functional connectivity in older adults depending on the induced current density. *Front. Aging Neurosci.* 13:725013. 10.3389/fnagi.2021.725013 34899266 PMC8662695

[B2] Alderson R. Patros C. Tarle S. Hudec K. Kasper L. Lea S. (2017). Working memory and behavioral inhibition in boys with ADHD: an experimental examination of competing models. *Child Neuropsychol.* 23 255–272. 10.1080/09297049.2015.1105207 26563880

[B3] Alizadehgoradel J. Nejati V. Movahed F. S. Imani S. Taherifard M. Mosayebi-Samani M. (2020). Repeated stimulation of the dorsolateral-prefrontal cortex improves executive dysfunctions and craving in drug addiction: a randomized, double-blind, parallel-group study. *Brain Stimul.* 13 582–593. 10.1016/j.brs.2019.12.028 32289681

[B4] Aron A. R. Poldrack R. A. (2006). Cortical and subcortical contributions to stop signal response inhibition: role of the subthalamic nucleus. *J. Neurosci.* 26 2424–2433. 10.1523/JNEUROSCI.4682-05.2006 16510720 PMC6793670

[B5] Aron A. R. Fletcher P. C. Bullmore E. T. Sahakian B. J. Robbins T. W. (2003). Stop-signal inhibition disrupted by damage to right inferior frontal gyrus in humans. *Nat. Neurosci.* 6 115–116. 10.1038/nn1003 12536210

[B6] Aron A. R. Herz D. M. Brown P. Forstmann B. U. Zaghloul K. (2016). Frontosubthalamic circuits for control of action and cognition. *J. Neurosci.* 36 11489–11495. 10.1523/jneurosci.2348-16.2016 27911752 PMC5125216

[B7] Bartholdy S. O’Daly O. G. Campbell I. C. Banaschewski T. Barker G. Bokde A. L. W. (2019). Neural correlates of failed inhibitory control as an early marker of disordered eating in adolescents. *Biol. Psychiatry* 85 956–965. 10.1016/j.biopsych.2019.01.027 31122340

[B8] Bender A. Filmer H. Dux P. (2017). Transcranial direct current stimulation of superior medial frontal cortex disrupts response selection during proactive response inhibition. *Neuroimage* 158 455–465. 10.1016/j.neuroimage.2016.10.035 27789261

[B9] Bikson M. Grossman P. Thomas C. Zannou A. Jiang J. Adnan T. (2016). Safety of transcranial direct current stimulation: evidence based update 2016. *Brain Stimul.* 9 641–661. 10.1016/j.brs.2016.06.004 27372845 PMC5007190

[B10] Brem A. K. Almquist J. N. F. Mansfield K. Plessow F. Sella F. Santarnecchi E. (2018). Modulating fluid intelligence performance through combined cognitive training and brain stimulation. *Neuropsychologia* 118 107–114. 10.1016/j.neuropsychologia.2018.04.008 29649503

[B11] Bruyer R. Brysbaert M. (2011). Combining speed and accuracy in cognitive psychology: is the Inverse Efficiency Score (IES) a better dependent variable than the mean Reaction Time (RT) and the Percentage Of Errors (PE)? *Psychol. Belgica* 51 5–13. 10.5334/pb-51-1-5

[B12] Chambers C. Bellgrove M. Stokes M. Henderson T. Garavan H. Robertson I. (2006). Executive “brake failure” following deactivation of human frontal lobe. *J. Cogn. Neurosci.* 18 444–455. 10.1162/089892906775990606 16513008

[B13] Chen T. Wang H. Wang X. Zhu C. Zhang L. Wang K. (2021). Transcranial direct current stimulation of the right dorsolateral prefrontal cortex improves response inhibition. *Int. J. Psychophysiol.* 162 34–39. 10.1016/j.ijpsycho.2021.01.014 33497765

[B14] Chen W. de Hemptinne C. Miller A. Leibbrand M. Little S. Lim D. (2020). Prefrontal-subthalamic hyperdirect pathway modulates movement inhibition in humans. *Neuron* 106 579–588.e3. 10.1016/j.neuron.2020.02.012 32155442 PMC7274135

[B15] Cohen J. (1992). A power primer. *Psychol. Bull.* 112 155–159. 10.1037/0033-2909.112.1.155 19565683

[B16] Congdon E. Mumford J. A. Cohen J. R. Adriana G. Turhan C. Poldrack R. A. (2012). Measurement and reliability of response inhibition. *Front. Psychol.* 3:37. 10.3389/fpsyg.2012.00037 22363308 PMC3283117

[B17] Cunillera T. Brignani D. Cucurell D. Fuentemilla L. Miniussi C. (2016). The right inferior frontal cortex in response inhibition: a tDCS-ERP co-registration study. *Neuroimage* 140 66–75. 10.1016/j.neuroimage.2015.11.044 26619787

[B18] Dagan M. Herman T. Harrison R. Zhou J. Giladi N. Ruffini G. (2018). Multitarget transcranial direct current stimulation for freezing of gait in Parkinson’s disease. *Mov. Disord.* 33 642–646. 10.1002/mds.27300 29436740 PMC5964604

[B19] Dambacher F. Schuhmann T. Lobbestael J. Arntz A. Brugman S. Sack A. (2015). No effects of bilateral tDCS over inferior frontal gyrus on response inhibition and aggression. *PLoS One* 10:e0132170. 10.1371/journal.pone.0132170 26161664 PMC4498781

[B20] Di Rosa E. Brigadoi S. Cutini S. Tarantino V. Dell’Acqua R. Mapelli D. (2019). Reward motivation and neurostimulation interact to improve working memory performance in healthy older adults: a simultaneous tDCS-fNIRS study. *Neuroimage* 202:116062. 10.1016/j.neuroimage.2019.116062 31369810 PMC7467146

[B21] Diamond A. (2013). Executive functions. *Annu. Rev. Psychol.* 64 135–168. 10.1146/annurev-psych-113011-143750 23020641 PMC4084861

[B22] Ehlis A. C. Haeussinger F. B. Gastel A. Fallgatter A. J. Plewnia C. (2016). Task-dependent and polarity-specific effects of prefrontal transcranial direct current stimulation on cortical activation during word fluency. *Neuroimage* 140 134–140. 10.1016/j.neuroimage.2015.12.047 26748077

[B23] Ehrhardt S. E. Filmer H. L. Wards Y. Mattingley J. B. Dux P. E. (2021). The influence of tDCS intensity on decision-making training and transfer outcomes. *J. Neurophysiol.* 125 385–397. 10.1152/jn.00423.2020 33174483

[B24] Enge S. Behnke A. Fleischhauer M. Kuttler L. Kliegel M. Strobel A. (2014). No evidence for true training and transfer effects after inhibitory control training in young healthy adults. *J. Exp. Psychol. Learn. Mem. Cogn.* 40 987–1001. 10.1037/a0036165 24707778

[B25] Enriquez-Geppert S. Huster R. Herrmann C. (2013). Boosting brain functions: improving executive functions with behavioral training, neurostimulation, and neurofeedback. *Int. J. Psychophysiol.* 88 1–16. 10.1016/j.ijpsycho.2013.02.001 23415793

[B26] Ester T. Kullmann S. (2021). Neurobiological regulation of eating behavior: evidence based on non-invasive brain stimulation. *Rev. Endocr. Metab. Disord.* Online ahead of print, 10.1007/s11154-021-09697-3 34862944 PMC9307556

[B27] Evans C. Bachmann C. Lee J. S. A. Gregoriou E. Ward N. Bestmann S. (2020). Dose-controlled tDCS reduces electric field intensity variability at a cortical target site. *Brain Stimul.* 13 125–136. 10.1016/j.brs.2019.10.004 31653475

[B28] Faul F. Erdfelder E. Lang A. Buchner A. (2007). G*Power 3: a flexible statistical power analysis program for the social, behavioral, and biomedical sciences. *Behav. Res. Methods* 39 175–191. 10.3758/bf03193146 17695343

[B29] Filmer H. L. Dux P. E. Mattingley J. B. (2014). Applications of transcranial direct current stimulation for understanding brain function. *Trends Neurosci.* 37 742–753. 10.1016/j.tins.2014.08.003 25189102

[B30] Fischer D. B. Fried P. J. Ruffini G. Ripolles O. Salvador R. Banus J. (2017). Multifocal tDCS targeting the resting state motor network increases cortical excitability beyond traditional tDCS targeting unilateral motor cortex. *Neuroimage* 157 34–44. 10.1016/j.neuroimage.2017.05.060 28572060 PMC12824506

[B31] Floden D. Stuss D. (2006). Inhibitory control is slowed in patients with right superior medial frontal damage. *J. Cogn. Neurosci.* 18 1843–1849. 10.1162/jocn.2006.18.11.1843 17069475

[B32] Friehs M. A. Brauner L. Frings C. (2021a). Dual-tDCS over the right prefrontal cortex does not modulate stop-signal task performance. *Exp. Brain Res.* 239 811–820. 10.1007/s00221-020-05995-5 33392696

[B33] Friehs M. A. Frings C. Hartwigsen G. (2021b). Effects of single-session transcranial direct current stimulation on reactive response inhibition. *Neurosci. Biobehav. Rev.* 128 749–765. 10.1016/j.neubiorev.2021.07.013 34271027

[B34] Fujiyama H. Tan J. Puri R. Hinder M. R. (2021). Influence of tDCS over right inferior frontal gyrus and pre-supplementary motor area on perceptual decision-making and response inhibition: a healthy ageing perspective. *Neurobiol. Aging* 109 11–21. 10.1016/j.neurobiolaging.2021.09.014 34634749

[B35] Gbadeyan O. McMahon K. Steinhauser M. Meinzer M. (2016). Stimulation of dorsolateral prefrontal cortex enhances adaptive cognitive control: a high-definition transcranial direct current stimulation study. *J. Neurosci.* 36 12530–12536. 10.1523/jneurosci.2450-16.2016 27974612 PMC6705663

[B36] Gowda S. M. Narayanaswamy J. C. Hazari N. Bose A. Chhabra H. Balachander S. (2019). Efficacy of pre-supplementary motor area transcranial direct current stimulation for treatment resistant obsessive compulsive disorder: a randomized, double blinded, sham controlled trial. *Brain Stimul.* 12 922–929. 10.1016/j.brs.2019.02.005 30808612

[B37] Gregoret L. Zamorano A. M. Graven-Nielsen T. (2021). Effects of multifocal transcranial direct current stimulation targeting the motor network during prolonged experimental pain. *Eur. J. Pain* 25 1241–1253. 10.1002/ejp.1743 33539582

[B38] Hannah R. Aron A. R. (2021). Towards real-world generalizability of a circuit for action-stopping. *Nat. Rev. Neurosci.* 22 538–552. 10.1038/s41583-021-00485-1 34326532 PMC8972073

[B39] Herrmann M. Plichta M. Ehlis A. Fallgatter A. (2005). Optical topography during a Go-NoGo task assessed with multi-channel near-infrared spectroscopy. *Behav. Brain Res.* 160 135–140. 10.1016/j.bbr.2004.11.032 15836908

[B40] Hill A. T. Rogasch N. C. Fitzgerald P. B. Hoy K. E. (2017). Effects of prefrontal bipolar and high-definition transcranial direct current stimulation on cortical reactivity and working memory in healthy adults. *Neuroimage* 152 142–157. 10.1016/j.neuroimage.2017.03.001 28274831

[B41] Hill A. T. Rogasch N. C. Fitzgerald P. B. Hoy K. E. (2018). Effects of single versus dual-site High-Definition transcranial direct current stimulation (HD-tDCS) on cortical reactivity and working memory performance in healthy subjects. *Brain Stimul.* 11 1033–1043. 10.1016/j.brs.2018.06.005 29936014

[B42] Hogeveen J. Grafman J. Aboseria M. David A. Bikson M. Hauner K. K. (2016). Effects of high-definition and conventional tDCS on response inhibition. *Brain Stimul.* 9 720–729. 10.1016/j.brs.2016.04.015 27198577

[B43] Holland R. Leff A. Josephs O. Galea J. Desikan M. Price C. (2011). Speech facilitation by left inferior frontal cortex stimulation. *Curr. Biol.* 21 1403–1407. 10.1016/j.cub.2011.07.021 21820308 PMC3315006

[B44] Hoogman M. Bralten J. Hibar D. P. Mennes M. Zwiers M. P. Schweren L. S. J. (2017). Subcortical brain volume differences in participants with attention deficit hyperactivity disorder in children and adults: a cross-sectional mega-analysis. *Lancet Psychiatry* 4 310–319. 10.1016/s2215-0366(17)30049-4 28219628 PMC5933934

[B45] Hoshi Y. Kobayashi N. Tamura M. (2001). Interpretation of near-infrared spectroscopy signals: a study with a newly developed perfused rat brain model. *J. Appl. Physiol.* 90 1657–1662. 10.1152/jappl.2001.90.5.1657 11299252

[B46] Hsu T. Y. Tseng L. Y. Yu J. X. Kuo W. J. Hung D. L. Tzeng O. J. (2011). Modulating inhibitory control with direct current stimulation of the superior medial frontal cortex. *Neuroimage* 56 2249–2257. 10.1016/j.neuroimage.2011.03.059 21459149

[B47] Hudak J. Blume F. Dresler T. Haeussinger F. Renner T. Fallgatter A. (2017). Near-infrared spectroscopy-based frontal lobe neurofeedback integrated in virtual reality modulates brain and behavior in highly impulsive adults. *Front. Hum. Neurosci.* 11:425. 10.3389/fnhum.2017.00425 28928644 PMC5591376

[B48] Hughes M. Fulham W. Johnston P. Michie P. (2012). Stop-signal response inhibition in schizophrenia: behavioural, event-related potential and functional neuroimaging data. *Biol. Psychol.* 89 220–231. 10.1016/j.biopsycho.2011.10.013 22027085

[B49] Huppert T. J. Diamond S. G. Franceschini M. A. Boas D. A. (2009). HomER: a review of time-series analysis methods for near-infrared spectroscopy of the brain. *Appl. Opt.* 48 D280–D298. 10.1364/ao.48.00d280 19340120 PMC2761652

[B50] Jacobson L. Javitt D. C. Lavidor M. (2011). Activation of inhibition: diminishing impulsive behavior by direct current stimulation over the inferior frontal gyrus. *J. Cogn. Neurosci.* 23 3380–3387. 10.1162/jocn_a_00020 21452949

[B51] Jasper H. H. (1958). Report of the committee on methods of clinical examination in electroencephalography. *Electroencephalogr. Clin. Neurophysiol.* 10 370–375. 10.1016/0013-4694(58)90053-1

[B52] Jurcak V. Tsuzuki D. Dan I. (2007). 10/20, 10/10, and 10/5 systems revisited: their validity as relative head-surface-based positioning systems. *Neuroimage* 34 1600–1611. 10.1016/j.neuroimage.2006.09.024 17207640

[B53] Kessler R. C. Adler L. Ames M. Demler O. Faraone S. Hiripi E. (2005). The World Health Organization adult ADHD Self-Report Scale (ASRS): a short screening scale for use in the general population. *Psychol. Med.* 35 245–256. 10.1017/s0033291704002892 15841682

[B54] Kuo H.-I. Bikson M. Datta A. Minhas P. Paulus W. Kuo M.-F. (2013). Comparing cortical plasticity induced by conventional and high-definition 4 x 1 Ring tDCS: a neurophysiological study. *Brain Stimul.* 6 644–648. 10.1016/j.brs.2012.09.010 23149292

[B55] Kwon Y. H. Kwon J. W. (2013a). Is transcranial direct current stimulation a potential method for improving response inhibition? *Neural Regen. Res.* 8 1048–1054. 10.3969/j.issn.1673-5374.2013.11.011 25206399 PMC4145879

[B56] Kwon Y. H. Kwon J. W. (2013b). Response inhibition induced in the stop-signal task by transcranial direct current stimulation of the pre-supplementary motor area and primary sensoriomotor cortex. *J. Phys. Ther. Sci.* 25 1083–1086. 10.1589/jpts.25.1083 24259920 PMC3818760

[B57] Li L. M. Violante I. R. Leech R. Hampshire A. Opitz A. McArthur D. (2019). Cognitive enhancement with Salience Network electrical stimulation is influenced by network structural connectivity. *Neuroimage* 185 425–433. 10.1016/j.neuroimage.2018.10.069 30385222 PMC6299257

[B58] Logan G. D. Cowan W. B. Davis K. A. (1984). On the ability to inhibit simple and choice reaction time responses: a model and a method. *J. Exp. Psychol. Hum. Percept. Perform.* 10 276–291. 10.1037//0096-1523.10.2.276 6232345

[B59] Lu H. Gong Y. Huang P. Zhang Y. Guo Z. Zhu X. (2020). Effect of repeated anodal HD-tDCS on executive functions: evidence from a pilot and single-blinded fNIRS study. *Front. Hum. Neurosci.* 14:583730. 10.3389/fnhum.2020.583730 33536886 PMC7847848

[B60] Maldonado T. Bernard J. A. (2021). The polarity-specific nature of single-session high-definition transcranial direct current stimulation to the cerebellum and prefrontal cortex on motor and non-motor task performance. *Cerebellum* 20 569–583. 10.1007/s12311-021-01235-w 33544371

[B61] Mayer J. T. Chopard G. Nicolier M. Gabriel D. Masse C. Giustiniani J. (2020). Can transcranial direct current stimulation (tDCS) improve impulsivity in healthy and psychiatric adult populations? A systematic review. *Progr. Neuropsychopharmacol. Biol. Psychiatry* 98:109814. 10.1016/j.pnpbp.2019.109814 31715284

[B62] Mizutani-Tiebel Y. Takahashi S. Karali T. Mezger E. Bulubas L. Papazova I. (2022). Differences in electric field strength between clinical and non-clinical populations induced by prefrontal tDCS: a cross-diagnostic, individual MRI-based modeling study. *Neuroimage Clin.* 34:103011. 10.1016/j.nicl.2022.103011 35487132 PMC9125784

[B63] Nagashima M. Monden Y. Dan I. Dan H. Tsuzuki D. Mizutani T. (2014). Acute neuropharmacological effects of atomoxetine on inhibitory control in ADHD children: a fNIRS study. *Neuroimage. Clin.* 6 192–201. 10.1016/j.nicl.2014.09.001 25379431 PMC4215398

[B64] Nikolin S. Loo C. K. Bai S. Dokos S. Martin D. M. (2015). Focalised stimulation using high definition transcranial direct current stimulation (HD-tDCS) to investigate declarative verbal learning and memory functioning. *Neuroimage* 117 11–19. 10.1016/j.neuroimage.2015.05.019 25987365

[B65] Nitsche M. A. Paulus W. (2000). Excitability changes induced in the human motor cortex by weak transcranial direct current stimulation. *J. Physiol.* 527(Pt. 3), 633–639. 10.1111/j.1469-7793.2000.t01-1-00633.x 10990547 PMC2270099

[B66] Nitsche M. Paulus W. (2001). Sustained excitability elevations induced by transcranial DC motor cortex stimulation in humans. *Neurology* 57 1899–1901. 10.1212/wnl.57.10.1899 11723286

[B67] Oldfield R. (1971). The assessment and analysis of handedness: the Edinburgh inventory. *Neuropsychologia* 9 97–113. 10.1016/0028-3932(71)90067-4 5146491

[B68] Orcioli-Silva D. Islam A. Baker M. R. Gobbi L. T. B. Rochester L. Pantall A. (2021). Bi-anodal transcranial direct current stimulation combined with treadmill walking decreases motor cortical activity in young and older adults. *Front. Aging Neurosci.* 13:739998. 10.3389/fnagi.2021.739998 34924993 PMC8681021

[B69] Pinti P. Tachtsidis I. Hamilton A. Hirsch J. Aichelburg C. Gilbert S. (2020). The present and future use of functional near-infrared spectroscopy (fNIRS) for cognitive neuroscience. *Ann. N. Y. Acad. Sci.* 1464 5–29. 10.1111/nyas.13948 30085354 PMC6367070

[B70] Pisoni A. Mattavelli G. Papagno C. Rosanova M. Casali A. G. Romero Lauro L. J. (2018). Cognitive enhancement induced by anodal tDCS drives circuit-specific cortical plasticity. *Cereb. Cortex* 28 1132–1140. 10.1093/cercor/bhx021 28184424

[B71] Reinhart R. M. G. Nguyen J. A. (2019). Working memory revived in older adults by synchronizing rhythmic brain circuits. *Nat. Neurosci.* 22 820–827. 10.1038/s41593-019-0371-x 30962628 PMC6486414

[B72] Rodrigo A. H. Domenico S. I. Ayaz H. Gulrajani S. Lam J. Ruocco A. C. (2014). Differentiating functions of the lateral and medial prefrontal cortex in motor response inhibition. *Neuroimage* 85(Pt. 1), 423–431. 10.1016/j.neuroimage.2013.01.059 23384524

[B73] Schneider N. Dagan M. Katz R. Thumm P. C. Brozgol M. Giladi N. (2021). Combining transcranial direct current stimulation with a motor-cognitive task: the impact on dual-task walking costs in older adults. *J. Neuroeng. Rehabil.* 18:23. 10.1186/s12984-021-00826-2 33526043 PMC7852224

[B74] Scholkmann F. Kleiser S. Metz A. J. Zimmermann R. Mata Pavia J. Wolf U. (2014). A review on continuous wave functional near-infrared spectroscopy and imaging instrumentation and methodology. *Neuroimage* 85(Pt. 1), 6–27. 10.1016/j.neuroimage.2013.05.004 23684868

[B75] Scholkmann F. Spichtig S. Muehlemann T. Wolf M. (2010). How to detect and reduce movement artifacts in near-infrared imaging using moving standard deviation and spline interpolation. *Physiol. Meas.* 31 649–662. 10.1088/0967-3334/31/5/004 20308772

[B76] Sehatpour P. Dondé C. Adair D. Kreither J. Lopez-Calderon J. Avissar M. (2021). Comparison of cortical network effects of high-definition and conventional tDCS during visuomotor processing. *Brain Stimul.* 14 33–35. 10.1016/j.brs.2020.11.004 33181350

[B77] Sharma M. Farahani F. Bikson M. Parra L. C. (2021). Weak DCS causes a relatively strong cumulative boost of synaptic plasticity with spaced learning. *Brain Stimul.* 15 57–62. 10.1016/j.brs.2021.10.552 34749007 PMC8816825

[B78] Steele V. Fink B. Maurer J. Arbabshirani M. Wilber C. Jaffe A. (2014). Brain potentials measured during a Go/NoGo task predict completion of substance abuse treatment. *Biol. Psychiatry* 76 75–83. 10.1016/j.biopsych.2013.09.030 24238783 PMC3984370

[B79] Stramaccia D. F. Penolazzi B. Sartori G. Braga M. Mondini S. Galfano G. (2015). Assessing the effects of tDCS over a delayed response inhibition task by targeting the right inferior frontal gyrus and right dorsolateral prefrontal cortex. *Exp. Brain Res.* 233 2283–2290. 10.1007/s00221-015-4297-6 25925996

[B80] Sun Q. L. Fang Y. H. Shi Y. Y. Wang L. F. Peng X. M. Tan L. W. (2021). Inhibitory top-down control deficits in schizophrenia with auditory verbal hallucinations: a Go/NoGo task. *Front. Psychiatry* 12:544746. 10.3389/fpsyt.2021.544746 34149464 PMC8211872

[B81] Thunberg C. Messel M. S. Raud L. Huster R. J. (2020). tDCS over the inferior frontal gyri and visual cortices did not improve response inhibition. *Sci. Rep.* 10:7749. 10.1038/s41598-020-62921-z 32385323 PMC7210274

[B82] Tsuzuki D. Jurcak V. Singh A. Okamoto M. Watanabe E. Dan I. (2007). Virtual spatial registration of stand-alone fNIRS data to MNI space. *Neuroimage* 34 1506–1518. 10.1016/j.neuroimage.2006.10.043 17207638

[B83] Valiengo L. Goerigk S. Gordon P. C. Padberg F. Serpa M. H. Koebe S. (2020). Efficacy and safety of transcranial direct current stimulation for treating negative symptoms in schizophrenia: a randomized clinical trial. *JAMA Psychiatry* 77 121–129. 10.1001/jamapsychiatry.2019.3199 31617873 PMC6802484

[B84] van Rooij D. Hoekstra P. J. Mennes M. von Rhein D. Thissen A. J. Heslenfeld D. (2015). Distinguishing adolescents with ADHD From their unaffected siblings and healthy comparison subjects by neural activation patterns during response inhibition. *Am. J. Psychiatry* 172 674–683. 10.1176/appi.ajp.2014.13121635 25615565 PMC4490085

[B85] Vaseghi B. Zoghi M. Jaberzadeh S. (2015). The effects of anodal-tDCS on corticospinal excitability enhancement and its after-effects: conventional vs. unihemispheric concurrent dual-site stimulation. *Front. Hum. Neurosci.* 9:533. 10.3389/fnhum.2015.00533 27242498 PMC4871166

[B86] Veit R. Schag K. Schopf E. Borutta M. Kreutzer J. Ehlis A. C. (2021). Diminished prefrontal cortex activation in patients with binge eating disorder associates with trait impulsivity and improves after impulsivity-focused treatment based on a randomized controlled IMPULS trial. *Neuroimage Clin.* 30:102679. 10.1016/j.nicl.2021.102679 34215149 PMC8102655

[B87] Verbruggen F. Logan G. D. (2008). Response inhibition in the stop-signal paradigm. *Trends Cogn. Sci.* 12 418–424. 10.1016/j.tics.2008.07.005 18799345 PMC2709177

[B88] Verbruggen F. Logan G. D. (2009). Models of response inhibition in the stop-signal and stop-change paradigms. *Neurosci. Biobehav. Rev.* 33 647–661. 10.1016/j.neubiorev.2008.08.014 18822313 PMC2696813

[B89] Verbruggen F. Aron A. R. Band G. P. H. Beste C. Bissett P. G. Brockett A. T. (2019). A consensus guide to capturing the ability to inhibit actions and impulsive behaviors in the stop-signal task. *Elife* 8:e46323. 10.7554/eLife.46323 31033438 PMC6533084

[B90] Villamar M. F. Volz M. S. Bikson M. Datta A. DaSilva A. F. Fregni F. (2013). Technique and considerations in the use of 4x1 ring high-definition transcranial direct current stimulation (HD-tDCS). *J. Vis. Exp.* 77:e50309. 10.3791/50309 23893039 PMC3735368

[B91] Wagenmakers E. J. Love J. Marsman M. Jamil T. Ly A. Verhagen J. (2018a). Bayesian inference for psychology. Part II: example applications with JASP. *Psychon. Bull. Rev.* 25 58–76. 10.3758/s13423-017-1323-7 28685272 PMC5862926

[B92] Wagenmakers E. J. Marsman M. Jamil T. Ly A. Verhagen J. Love J. (2018b). Bayesian inference for psychology. Part I: theoretical advantages and practical ramifications. *Psychon. Bull. Rev.* 25 35–57. 10.3758/s13423-017-1343-3 28779455 PMC5862936

[B93] Wagenmakers E. J. Wetzels R. Borsboom D. van der Maas H. L. (2011). Why psychologists must change the way they analyze their data: the case of psi: comment on Bem (2011). *J. Pers. Soc. Psychol.* 100 426–432. 10.1037/a0022790 21280965

[B94] Wang Y. Liu L. Zhang Y. Wei C. Xin T. He Q. (2021). The neural processing of vocal emotion after hearing reconstruction in prelingual deaf children: a functional near-infrared spectroscopy brain imaging study. *Front. Neurosci.* 15:705741. 10.3389/fnins.2021.705741 34393716 PMC8355545

[B95] Weidler C. Habel U. Wallheinke P. Wagels L. Hofhansel L. Ling S. (2020). Consequences of prefrontal TDCS on inhibitory control and reactive aggression. *Soc. Cogn. Affect. Neurosci.* 17 120–130. 10.1093/scan/nsaa158 33227131 PMC8824612

[B96] Whelan R. Conrod P. Poline J. Lourdusamy A. Banaschewski T. Barker G. (2012). Adolescent impulsivity phenotypes characterized by distinct brain networks. *Nat. Neurosci.* 15 920–925. 10.1038/nn.3092 22544311

[B97] Wu D. Zhang P. Liu N. Sun K. Xiao W. (2021a). Effects of high-definition transcranial direct current stimulation over the left fusiform face area on face view discrimination depend on the individual baseline performance. *Front. Neurosci.* 15:704880. 10.3389/fnins.2021.704880 34867146 PMC8639859

[B98] Wu D. Zhou Y. Xu P. Liu N. Sun K. Xiao W. (2021b). Initial performance modulates the effects of cathodal transcranial direct current stimulation (tDCS) over the right dorsolateral prefrontal cortex on inhibitory control. *Brain Res.* 1774:147722. 10.1016/j.brainres.2021.147722 34774867

[B99] Xu P. Wu D. Chen Y. Wang Z. Xiao W. (2020). The effect of response inhibition training on risky decision-making task performance. *Front. Psychol.* 11:1806. 10.3389/fpsyg.2020.01806 32793080 PMC7393991

[B100] Yang M. Yang Z. Yuan T. Feng W. Wang P. (2019). A systemic review of functional near-infrared spectroscopy for stroke: current application and future directions. *Front. Neurol.* 10:58. 10.3389/fneur.2019.00058 30804877 PMC6371039

[B101] Yaqub M. A. Woo S.-W. Hong K.-S. (2018). Effects of HD-tDCS on resting-state functional connectivity in the prefrontal cortex: an fNIRS study. *Complexity* 129 1–8. 10.1155/2018/1613402

[B102] Yavari F. Jamil A. Samani M. M. Vidor L. P. Nitsche M. A. (2018). Basic and functional effects of transcranial Electrical Stimulation (tES)-An introduction. *Neurosci. Biobehav. Rev.* 85 81–92. 10.1016/j.neubiorev.2017.06.015 28688701

[B103] Ye J. Tak S. Jang K. Jung J. Jang J. (2009). NIRS-SPM: statistical parametric mapping for near-infrared spectroscopy. *Neuroimage* 44 428–447. 10.1016/j.neuroimage.2008.08.036 18848897

[B104] Yeh C. B. Gau S. S. Kessler R. C. Wu Y. Y. (2008). Psychometric properties of the Chinese version of the adult ADHD Self-report scale. *Int. J. Methods Psychiatr. Res.* 17 45–54. 10.1002/mpr.241 18286465 PMC6878254

[B105] Yu J. Tseng P. Hung D. L. Wu S. W. Juan C. H. (2015). Brain stimulation improves cognitive control by modulating medial-frontal activity and preSMA-vmPFC functional connectivity. *Hum. Brain Mapp.* 36 4004–4015. 10.1002/hbm.22893 26248582 PMC6869595

[B106] Zarahn E. Rakitin B. Abela D. Flynn J. Stern Y. (2007). Age-related changes in brain activation during a delayed item recognition task. *Neurobiol. Aging* 28 784–798. 10.1016/j.neurobiolaging.2006.03.002 16621168

[B107] Zhang T. Y. Zhang J. Q. Huang J. X. Zheng Z. Wang P. (2021). Neural activation via acupuncture in patients with major depressive disorder: a functional near-infrared spectroscopy study. *Front. Psychiatry* 12:669533. 10.3389/fpsyt.2021.669533 34867499 PMC8632864

[B108] Zhao X. Chen L. Maes J. (2018). Training and transfer effects of response inhibition training in children and adults. *Dev. Sci.* 21:1. 10.1111/desc.12511 27966279

[B109] Zhou J. Manor B. Yu W. Lo O. Y. Gouskova N. Salvador R. (2021). Targeted tDCS mitigates dual-task costs to gait and balance in older adults. *Ann. Neurol.* 90 428–439. 10.1002/ana.26156 34216034 PMC8434977

[B110] Zhuo L. Zhao X. Zhai Y. Zhao B. Tian L. Zhang Y. (2022). Transcutaneous electrical acupoint stimulation for children with attention-deficit/hyperactivity disorder: a randomized clinical trial. *Transl. Psychiatry* 12:165. 10.1038/s41398-022-01914-0 35449191 PMC9022403

