# Supplementary material for: Multitarget high-definition transcranial direct current stimulation improves response inhibition more than single-target high-definition transcranial direct current stimulation in healthy participants
Source: Front Neurosci. 2022 Jul 29;16:905247. doi: 10.3389/fnins.2022.905247 (PMC9372262; doi:10.3389/fnins.2022.905247)
Supplement: Supplementary file 2 [file Image_1.pdf]

Supplementary Material

1 Supplementary Data

Supplementary Datasheet 1. The data of △deoxy-Hb and △total-Hb in the pre-SMA ROI and rIFG ROI.

2 Supplementary Figures

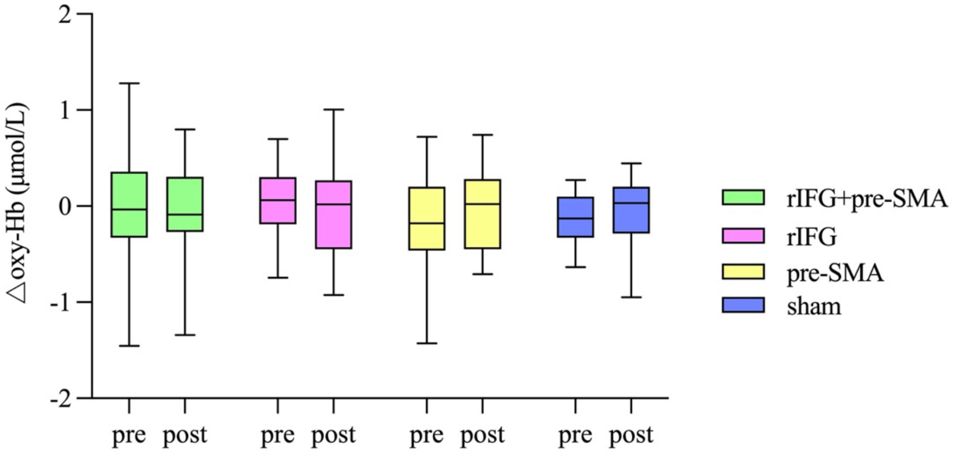

Supplementary Figure 1. Changes in △oxy-Hb from pretest to posttest in the rIFG ROI. Boxes extend from the 25th to 75th percentiles with a horizontal line representing the median. Whiskers show the min to max values.
